# Radiological tarsal bone morphology in adolescent age of congenital clubfeet treated with the Ponseti method

**DOI:** 10.1186/s12891-021-04193-w

**Published:** 2021-04-06

**Authors:** Johannes Hamel, Hubert Hörterer, Norbert Harrasser

**Affiliations:** 1 Schön Klinik München Harlaching, Fachzentrum für Fuß- und Sprunggelenkchirurgie, Harlachinger Str. 51, 81547 Munich, Germany; 2 Klinik für Allgemeine- Unfall- und Wiederherstellungschirurgie, Klinikum der Universität München, LMU München, Nussbaumstrasse 20, 80336 München, Germany; 3 Klinikum rechts der Isar, Department of Orthopedics and Sports Orthopedics, Technical University Munich, Ismaninger Str. 22, 81675 Munich, Germany

**Keywords:** Flat top talus, Idiopathic clubfoot, Talus development, Tarsal bones

## Abstract

**Background:**

Major abnormalities of tarsal bone shape after surgical clubfoot treatment are well known from the literature. The Ponseti method has gained widespread acceptance in primary treatment of congenital clubfeet. Despite the longtime experience, data regarding the development of tarsal bones after this treatment are still rare. The aim of the study was therefore to evaluate radiographic parameters describing tarsal bone shape of clubfeet after Ponseti treatment and compare them to age-matched healthy feet.

**Methods:**

Twenty two consecutive severe clubfeet in 14 patients were investigated by repeated diagnostic ultrasound during the corrective process. Extent and temporal course of correction were documented with standardized X-rays after a mean follow-up of 12 years (between the ages of 10–14 years) and compared to a group of age-matched normal feet.

**Results:**

Reliability testing for all X-ray parameters showed good to excellent results. In comparison to the control group, all parameters except the radius of the trochlea (RT) were significantly altered in clubfeet with highest differences shown for the anterior talar motion segment (ATM), talonavicular coverage (TNC) and the talometatarsal index (TMT-Index). Differentiation between minor and major deformities showed significant differences only for the front tarsal index (FTI).

**Conclusions:**

Tarsal bone shape of clubfeet treated by the Ponseti method differs significantly from normal feet. One of the most pronounced and clinically relevant difference of the clubfoot talus compared to the normal talus is the markedly reduced anterior talar motion segment.

## Background

The Ponseti method is a well-established treatment regimen for congenital clubfeet with high rates of clinical corrections [1, 2]. Early clubfoot recurrences after Ponseti treatment, often caused by persistent muscular imbalances and especially low brace compliance, are not uncommon and can also be effectively treated with this method [3]. However, apart from studies by Ponseti himself, there is only limited knowledge about the natural development of tarsal bones after Ponseti treatment [4]. In this context, some authors reported the involvement of lower limbs genetic mechanisms and possible influence on the development of tarsal bones [5]. Radiographic and sonographic studies on the development of tarsal bone shape in treated clubfeet revealed marked differences from normal feet but observed also some methodological difficulty in detecting morphological differences with these imaging modalities [6–10]. Additionally, it is unknown whether severe tarsal bone deformities, often observed after primary surgical therapy of clubfeet, occur or persist after the Ponseti treatment [11–14]. Clinical experience with residual clubfoot deformities in adult patients shows that in many cases the condition of the ankle will determine the long-term clinical outcome [15–17]. In this context, supramalleolar valgus misgrowth and dysplasia of the talus with a significantly reduced or flattened articular surface of the trochlea tali (“flat top talus”) have often been observed after initial surgical treatment of clubfeet and were associated with poor clinical outcome [12, 14, 18]. Comparable deformities might also affect the outcome after therapy with the Ponseti method. Therefore, the aim of the present study was to investigate the frequency and extent of such changes in a defined group of patients after primary Ponseti therapy and compare the morphological changes to an aged-matched control group of normal feet. We hypothesized that the shape of tarsal bones in clubfeet treated with the Ponseti method might significantly differ from their normal counterparts.

## Patients and methods

**Fig. 1:**
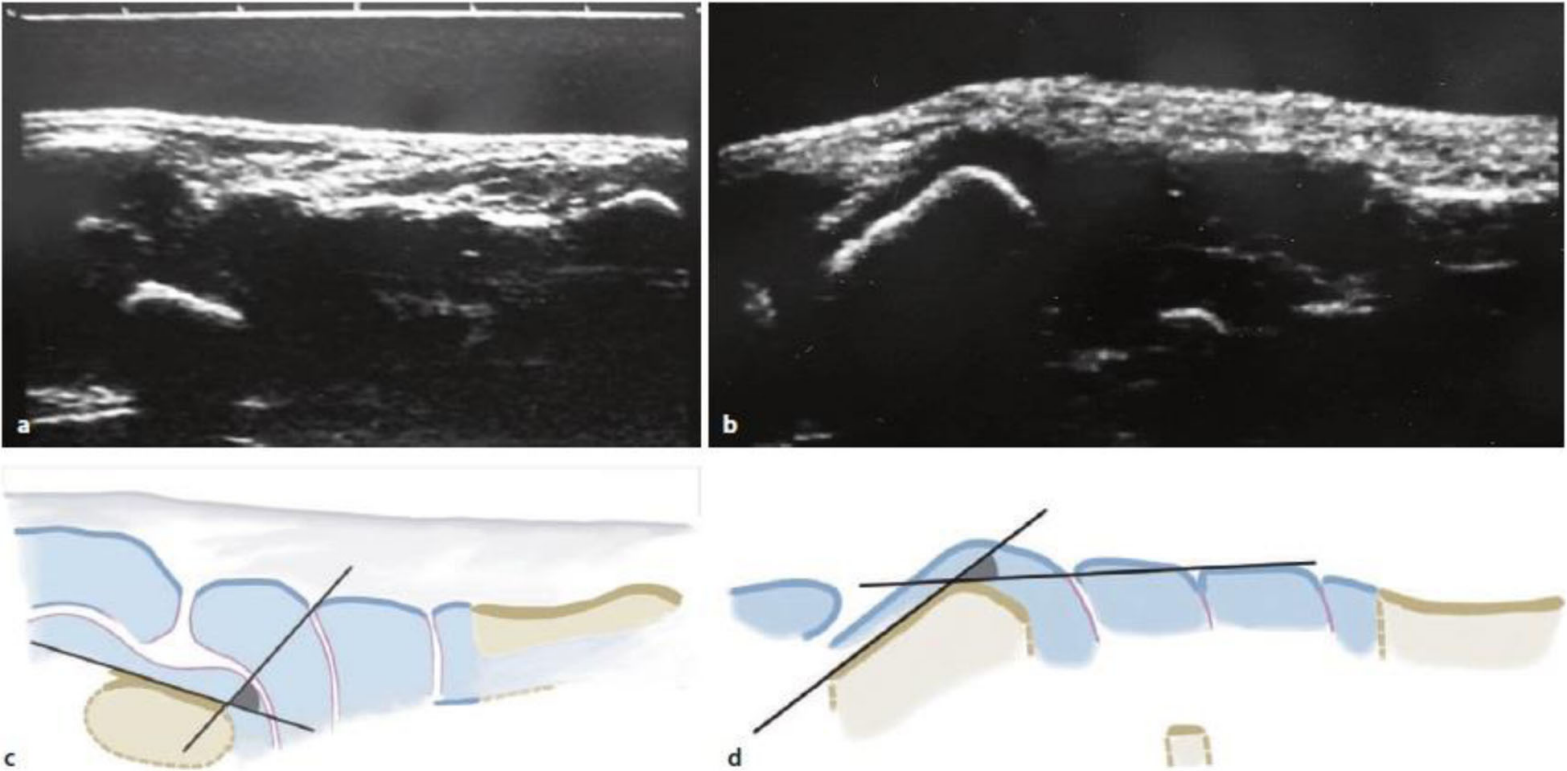
Sonographic transverse section of an uncorrected clubfoot at the age of 5 months (**a, c**) with a TnCE angle of approx. + 75 ° and healthy contralateral side (**b, d**) with TnCE angle of approx. -30 °

The retrospective study was approved by our hospital’s institutional review board. Seventeen patients (male:female = 12:5) with 25 idiopathic clubfeet and high initial misalignment were evaluated during and after Ponseti treatment. Inclusion criteria were: Patients with no former treatment and an initial TnCE-angle [19] of above + 30° (Fig. 1), corresponding roughly to grade 3 or 4 in the Dimeglio classification system. Exclusion criteria were: First referral after the first month of life, mild forms with tarsal malalignment below + 30° TnCE-angle, atypical or neurological forms of clubfoot, all patients that did not complete primary treatment and aftercare at the first authors‘ institution up to the fourth birthday, low brace compliance and early cessation of bracing. All serial castings and operative tenotomies were performed by the first author of the study. Treatment consisted of long leg casts until the plateau was reached (5 to 8 casts), tenotomy in all of the cases and three months of retention in the foot abduction brace for 23 h a day and afterwards at night- and resting times up to the fourth birthday of the child. Initial clinical correction was achieved in all the cases, however the time to correction was significantly longer from a sonographic point of view. Functional sonography during the correction process (reduction phase) and up to the age of 4–7 years (until the end of the retention phase) showed a complete correction of the medial tarsal region based on sonographic criteria. The mobility of the hindfoot during eversion was always restricted in comparison to normal feet. Twenty two out of 25 clubfeet (88 %, 14 patients, 8 with bilateral deformity) were available for final follow-up after a mean of 12.0 years (range: 10.5–14.7 years). In 8 cases (36.4 %) an anterior tibialis transfer with dorsal release due to muscular imbalance and impending recurrence was performed between the ages of 4–6 years. In 7 cases (31.8 %) the restriction of ankle dorsiflexion < 10° was treated temporarily with the use of a dynamic night-splint. No further surgical or non-surgical interventions were necessary.

**Fig. 2:**
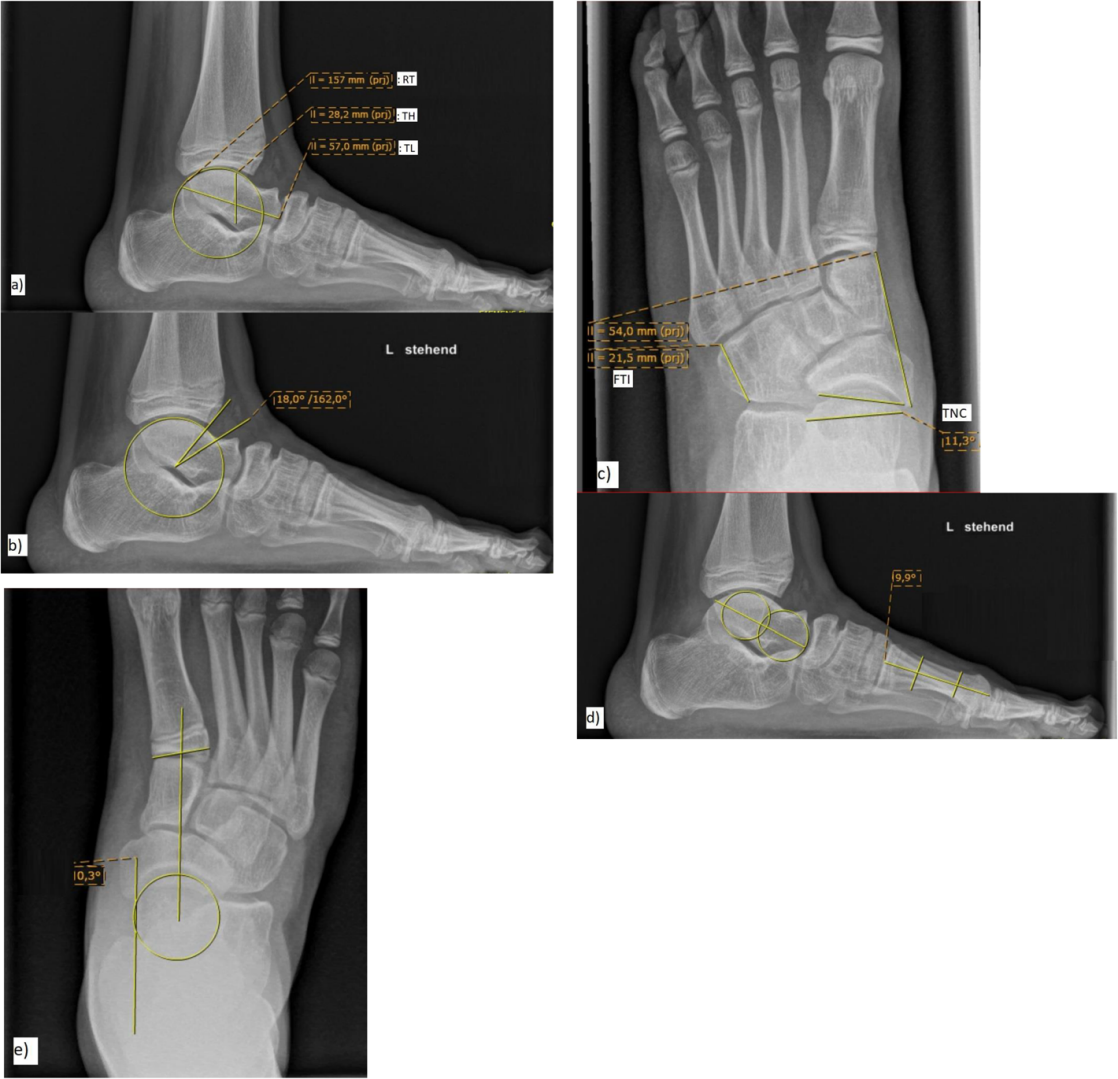
**a-e**: Measurement of talar length (TL; distance from posterior extremity to the talar head), radius of the trochlea tali (RT), talar height (TH; perpendicular distance from the tip of the lateral talar process inferiorly to the trochlea tali superiorly) (**a**), “anterior talar motion segment” (ATM; angular segment available for tibiotalar dorsiflexion in the plantigrade foot position between most anterior part of the trochlea tali and anterior distal tibiaplafond) (**b**), “talonavicular coverage” (TNC; angle between articular surface of the talus and the navicular bone), “front tarsal index” (FTI; relation of medial cuneiform-navicular distance and cuboid distance) (**c**), “Meary-Angle” (MA; angle between long axis of the talus and the first metatarsal bone) (**d**), “Talus-Metatarsal-base-I angle” (TMTB-I; angle between long axis of the talus and a line from the middle of the talar head to the middle of the first metatarsal base) (**e**)

Standardized X-rays (ankle a.-p., foot lateral and d.-p.) were performed under full weight-bearing conditions. In order to obtain exact lateral views of the ankle joint to precisely measure parameters of the talus, the rotation of the ankle had to be adjusted individually. Eight parameters characterizing the shape of the tarsal bones were measured/calculated as previously described (Fig. 2a-e) [13, 14, 20]: Talar length (TL), the radius of the trochlea tali (RT) and the talar height (TH) were measured in the lateral view of the foot and the ratio radius/length (RT/LT) was calculated accordingly. To describe the angular segment available for tibiotalar dorsiflexion the “anterior talar motion segment” (ATM) was measured in the lateral view. The talonavicular relationship was determined from the dorsoplantar view of the foot using the angle of the talar and navicular articular surface (talonaviculare coverage, TNC). The “front tarsal index” (FTI) denotes the relation of the medial and lateral length of the front tarsus. The Talometatarsal-Index (TMT-Index) was calculated as the sum of the Meary-Angle (MA) in the lateral view of the foot and the Talometatarsal-base-I angle (TMTB-I-angle) in the d.-p. view of the foot. It describes a measure of the overall position of the rearfoot complex and the medial tarsal ray. In addition, the a.-p. view of the ankle was assessed for any supramalleolar frontal deformity.

Depending on the severity of the initial deformity the study group was subsequently divided for further analysis: Eight cases (subgroup 1) with “minor”, 14 cases (subgroup 2) with “major” deformity. “Minor” deformity was defined as: initial TnC_E_ angle < + 50° and/or sonographically detected rapid correction after initial casting; if these criteria were not fulfilled the deformity was classified as “major”.

Due to ethical restrictions X-rays cannot be performed in healthy children without any foot disorders. Hence, the age-matched control group (mean age: 12,1 years, range: 10.2–14.8 years) was recruited retrospectively by screening records of patients who presented at our clinic for isolated forefoot disorders (e.g. Hallux valgus, Brachymetatarsia, Mb. Koehler II, Metatarsalgia) and no hindfoot pathologies (e.g. flatfoot, cavovarus foot). All patients had no prior foot surgery.

In order to obtain intraobserver reliability, repeated measurements were conducted by the first author (“A”; foot and ankle surgeon) after 3 months from the initial measurement. The senior author (“B”; foot and ankle surgeon) performed measurements of the same subset of data and results were compared among each other to obtain interobserver reliability.

### Statistical analysis

Prior to analysis, normal distribution was verified using Kolmogorov-Smirnov testing. The two-tailed t-test was performed to determine significant differences between the means of the groups. Statistical significance was assumed for all *p*-values < 0.05. Intra- and interobserver reliability was determined using Pearson’s correlation coefficient (PCC). Reliability was classified as minimal (PCC ≤ 0.25), low (0.26 < PCC < 0.5), moderate (0.5 ≤ PCC < 0.7), high (0.7 ≤ PCC < 0.9), and excellent (PCC ≥ 0.9) [21]. All analyses were performed with GraphPad Prism 5 (GraphPad Software, Inc., La Jolla, USA).

## Results

X-ray parameters of study and control groups with corresponding *p*-values and ratio clubfoot/controls are given in Table 1. In comparison to the control group, all parameters except the radius of the trochlea (RT) were significantly altered in clubfeet. Analysis of single parameters showed a significantly greater front tarsal index (FTI) in clubfeet compared to healthy feet, indicating relative over length of the medial column at the level of the midfoot. On the other hand talar length (TL) and talar height (TH) were significantly smaller in clubfeet indicating a tendency towards a flat top talus in these cases. Correspondingly to this finding, the ratio of RT/TL was greater in clubfeet. This configuration of the bone shape will result in a certain loss of motion towards extension of the ankle joint, which was further confirmed by the significant decrease of the anterior talar motion segment (ATM). In accordance to the talonavicular joint configuration with a higher TN-coverage the TMT-Index showed values indicating a straighter orientation of the medial column in clubfeet compared to the controls.

**Table 1:** X-ray parameters (mean ± standard deviation) of controls and clubfeet;* TL* talar length, *RT *radius of trochlea tali, *TH* talar height, *ATM* anterior talar motion segment, *TNC* talonavicular coverage, *FTI* front tarsal index, *TMT-Index* Talometatarsal-Index

	Controls (*n* = 22)	Clubfeet (*n* = 22)	Ratio (clubfeet/controls)	*p*-value (clubfeet/controls)
**TL (mm)**	55,3 (± 4,1)	52,8 (± 3,6)	0,95	< 0,05
**RT (mm)**	21,7 (± 2,2)	22,8 (± 2,5)	1,05	0,16
**Ratio-RT/TL**	0,39 (± 0,03)	0,43 (± 0,04)	1,10	< 0,05
**TH (mm)**	32,3 (± 2,5)	27,2 (± 2,7)	0,84	< 0,05
**ATM (°)**	29,1 (± 6,3)	15,4 (± 4,9)	0,53	< 0,05
**TN-Coverage (°)**	-17,0 (± 7,3)	3,0 (± 10,1)	0,15	< 0,05
**FTI**	2,0 (± 0,2)	2,6 (± 0,5)	1,32	< 0,05
**TMT-Index (°)**	-18,0 (± 7,1)	-5,0 (± 14,4)	0,28	< 0,05

Differentiation between minor and major deformities is given in Table 2. Even though clear tendencies between the amount of initial deformities and X-ray parameters were found, statistical significant differences between subgroup 1 and 2 were only shown for FTI. Nevertheless, analysis of radiographic parameters with calculated ratio subgroup 1/subgroup 2 showed exactly the same direction as the ratio control group/clubfeet (Table 1).

**Table 2:** X-ray parameters (mean ± standard deviation) of minor/major deformities within the clubfoot-group; *TL* talar length, *RT* radius of trochlea tali, *TH* talar height, *ATM *anterior talar motion segment, *TNC* talonavicular coverage, *FTI* front tarsal index, *TMT-Index *Talometatarsal-Index

	Subgroup 1 (minor, *n* = 8)	Subgroup 2 (major, *n* = 14)	Ratio (subgroup 1/controls)	Ratio (subgroup 2/controls)	*p*-value (subgroup 1/subgroup 2)
**TL (mm)**	54,6 (± 2,9)	51,7 (± 3,6)	0,99	0,94	0,07
**RT (mm)**	22,6 (± 2,7)	22,9 (± 2,4)	1,04	1,05	0,84
**Ratio-RT/TL**	0,41 (± 0,03)	0,44 (± 0,04)	1,05	1,13	0,09
**TH (mm)**	28,4 (± 2,7)	26,6 (± 2,4)	0,88	0,82	0,14
**ATM (°)**	15,8 (± 3,7)	15,1 (± 5,4)	0,54	0,52	0,79
**TN-Coverage (°)**	-2,4 (± 13,0)	6,1 (± 6,0)	0,14	-0,36	0,06
**FTI**	2,2 (± 0,2)	2,8 (± 0,5)	1,10	1,40	< 0,05
**TMT-Index (°)**	-6,0 (± 16,7)	-3,8 (± 12,8)	0,34	0,22	0,74

Reliability testing for all X-ray parameters showed good to excellent intra- and interobserver reliability (Table 3).

**Table 3:** Intra-/interobserver reliability testing (Pearson correlation coefficient)

Parameters	Intraobserver reliability	Interobserver reliability
	**Observer A**	**Observer A and B**
TL (mm)	0.90	0.85
RT (mm)	0.91	0.92
TH (mm)	0.83	0.89
ATM (°)	0.87	0.83
TN-Coverage (°)	0.75	0.87
FTI	0.90	0.89
TMT-Index (°)	0.91	0.92

Relevant supramalleolar valgus deformities, defined as TASA (“tibial anterior surface angle”; normal: 91–93°[22]) > 99°, were detected in 4 cases (18 %). In two of these cases the deformity was rather small (TASA 100° and 102°). One of the cases was a patient with bilateral severe clubfeet and persistent equinus foot despite tenotomy of the Achilles tendon. At the age of 14 months an open bilateral dorsal release was performed and showed an accessory soleus muscle as a potential cause.

## Discussion

In the present study we evaluated the development of tarsal bones in the long term after treatment of clubfeet with the Ponseti method. Unsurprisingly, we found that tarsal bones in treated clubfeet were deformed compared to normal feet. Nevertheless, several radiological parameters describing the shape and interaction of tarsal bones showed satisfactory treatment success. Additionally, it was found that radiological outcome does not necessarily depend on the severity of deformity prior treatment initiation. We are not aware of any other comparable studies on tarsal shape development in adolescence or adulthood.

Ponseti was the first to compare X-rays of both feet in two planes of 32 patients with unilateral clubfoot after completion of growth and found qualitatively very similar changes to the patient group presented here [4]. In the quantitative analysis, however, Ponseti and Co-authors considered different parameters than in the present study, e.g. no ankle a.-p. view was performed. Additionally, the treatment regimen varied compared to current standards, e.g. the retention phase was considerably shorter than the 4 years recommended nowadays.

The shape of tarsal bones in severe idiopathic clubfeet treated with the Ponseti method differs from that of unaffected feet of the same age (Table 1). In comparison to the literature on results based on previous primary-operative treatment concepts, however, these differences appear to be significantly smaller. In particular, no cases of “small dome talus” or “flat top talus” were observed, as was the case in other studies reporting up to 22 % of these changes after dorsomedial release [13]. In the present study, talar length was only minimally reduced to an average of 95 % compared to the control group. On the other hand, talar height was highly reduced to 84 % compared to normal feet. All morphological differences of clubfeet compared to normal feet appeared to be more pronounced in the more severely affected patients of the present study. However, significant differences could only be found for the FTI. This might also be explainable by the small number of patients. Summarizing the aforementioned morphological data, the talus in clubfeet after Ponseti treatment is slightly shortened with only a very slight increase in the trochlear radius. This coincides with magnetic resonance imaging findings in small children after Ponseti primary therapy [23]. The height of the trochlea tali appears to be significantly reduced compared to healthy feet. The shape of the subtalar joint and the configuration of the lateral talar process (e.g. dysplasia) could influence this parameter as already reported [4]. However, this was not examined in detail in this study. The reason for the comparatively favorable formation of the trochlea tali after Ponseti primary therapy could be caused by the amount of free dorsiflexion after tenotomy, which was achieved very early in the course of treatment (around the age of 6–13 weeks). In the previously common primary surgical treatment concepts, this was usually achieved not before 6 months in conjunction with the surgical intervention.

The differences regarding the “anterior talar motion segment” were clearly detectable, corresponding to the limitation in dorsiflexion often observed in clubfoot patients. Numerous factors are included in this parameter, e.g. talar height, radius of the trochlea tali, shape of the talus neck (“off-set”), but also the talus-floor angle and others. Some authors stated the significantly reduced shape of the talus-neck transition to be the main cause for persistent restriction in dorsiflexion of the ankle in toddlers treated with the Ponseti method [23].

**Fig. 3:**
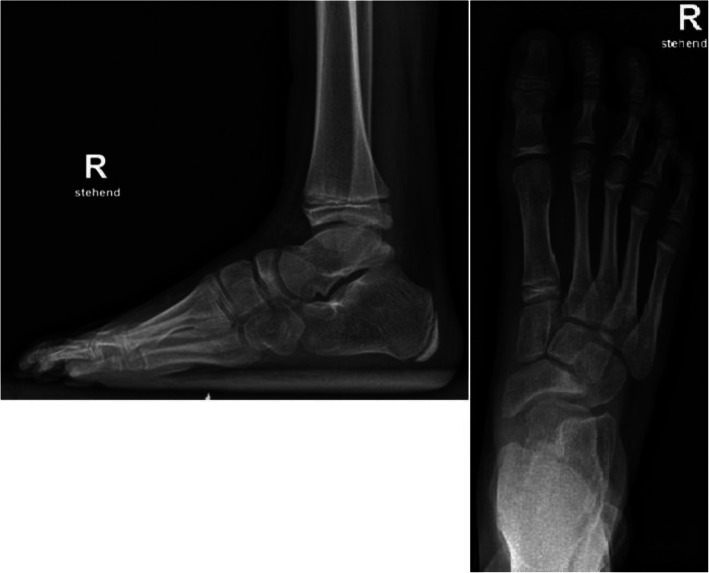
Images of an 11-year-old patient with initially severe clubfoot and comparatively pronounced tarsal shape changes. Ratio RT / TL = 0.47. Incomplete talonavicular reduction (TN coverage + 13 °) with slight rounding of the lateral talus head, flattening of the lateral navicular bone and a high FTI (3.2) can be seen in the dorsoplantar image

A common finding after Ponseti treatment is an incomplete talonavicular repositioning and mobility in the transverse plane [4]. As a sort of compensation mechanism a wedge-shaped navicular bone with lateral flattening and lateral deviation in the naviculo-cuneiform joint-line can be observed (Fig. 3). This tarsal configuration was often found in our patients and consequently led to significantly altered talonavicular position angles and a FTI that deviated significantly from healthy feet.

The TMT index [24] was used in the present study to describe the foot position in general. It showed clear differences between clubfeet and healthy feet. This demonstrates the reduced flexibility in eversion of the rear- and midfoot in idiopathic clubfoot.

Several authors described rates up to 66 % of supramalleolar valgus deformities after primary surgical treatment of clubfeet [12, 18]. The cause of this growth disorder is not fully understood; it is observed frequently in over-correction conditions [25]. In the patient group examined here, valgus deformities occurred only in 4 cases (18 %), of which one patient represents a special case within the study group due to persistent equinus foot position after tenotomy of the Achilles tendon as a result of an accessory soleus muscle found after open dorsal release. The low rates of supramalleolar valgus deformity after Ponseti treatment in our study shows a clear advantage of this treatment in comparison to primary operative therapy concepts.

The main limitations of the present study are the small number of patients which is mainly caused by the single-center study design, and the lack of clinical assessment. Nevertheless, the sample size is in accordance with other studies on this topic [2, 4]. The present investigation meant to focus primarily on radiological parameters after Ponseti treatment. Even though it is well accepted, that reduction of the deformity correlates with clinical outcome after clubfoot treatment, we did not acquire clinical outcome data in our cohort. Nevertheless, this has to do with form and function. If correction of anatomy is incomplete, limitations in foot function are likely to persist. Another weakness of the study is the fact that in most cases the development of the foot was not yet completed at final follow-up. The patients’ sex with the known temporally different maturation of the feet was neither considered in the clubfoot group nor in the normal group. Patients were included in the control group due to foot problems others than clear rear- or midfoot disorders. These feet were considered “normal” even though some foot problems were present. However, radiographic imaging of children can only be performed with strict ethical considerations making recruitment of control groups for study purposes without any foot disorders very difficult or rather impossible.

## Conclusions

In summary it can be stated that the development of tarsal skeletal elements in severe clubfeet treated by the Ponseti primary treatment concept and later additional limited surgery if indicated is favourable in comparison to descriptions of other treatment methods in literature. The well known primary dysplastic shape of the talus seems to recover largely, however does not reach completely the normal values of age-matched adolescents. Especially the radius of the talar surface in the sagittal profile is not significantly different and talar length and talar height are only slightly but nevertheless significantly reduced. The most pronounced difference of the clubfoot talus compared to normal talar shape is the markedly reduced anterior talar motion segment. Severe supramalleolar valgus malgrowth was observed only in one bilaterally affected child with a course complicated by an accessory soleus muscle. A certain degree of the primary talonavicular malalignment in the transversal plane persists to adolescent age and is compensated by adaptive changes in the anterior tarsal region. All these anomalies seem to be related to the primary severity of clubfoot, however this was significant not for all examined parameters in the limited number of patients included into the study.

## Data Availability

The datasets generated during and analyzed during the current study are published in the manuscript. Single values are available from the corresponding author on reasonable request.

## Abbreviations

TL: Talar length
RT: Radius of trochlea tali
TH: Talar height
ATM: Anterior talar motion segment
TNC: Talonavicular coverage
FTI: Front tarsal index
TMT-Index: Talometatarsal-Index
